# Development and evaluation of a test strip for the rapid detection of antibody against equine infectious anemia virus

**DOI:** 10.1007/s00253-023-12980-9

**Published:** 2024-01-08

**Authors:** Zenan Zhang, Kui Guo, Xiaoyu Chu, Mingru Liu, Cheng Du, Zhe Hu, Xiaojun Wang

**Affiliations:** 1 State Key Laboratory for Animal Disease Control and Prevention, Harbin Veterinary Research Institute, the Chinese Academy of Agricultural Sciences, Harbin, China; 2 Institute of Western Agriculture, the Chinese Academy of Agricultural Sciences, Changji, China; 3 WOAH Reference Laboratory for Equine Infectious Anemia, Harbin Veterinary Research Institute, the Chinese Academy of Agricultural Sciences, Harbin, China; 4 Shenzhen Lvshiyuan Biotechnology Co., Shenzhen, China

**Keywords:** Equine infectious anemia, Serological test, Colloidal gold immunochromatographic test, p26 and gp45, Strip

## Abstract

**Abstract:**

Equine infectious anemia (EIA) is a contagious disease of horses caused by the equine infectious anemia virus (EIAV). The clinical signs at the acute phase include intermittent high fever, thrombocytopenia, hemorrhage, edema, and anemia. The clinical signs at chronic and relapsing subclinical levels include emaciation and progressive weakness. Surviving horses become lifelong carriers because of the integration of the viral genome into that of the host, and these horses can produce and transmit the virus to other animals. This increases the difficulty of imposing practical control measures to prevent epidemics of this disease. Serological tests measuring the antibodies in equine sera are considered to be a reliable tool for the long-term monitoring of EIA. However, the standard serological tests for EIV either have low sensitivity (e.g., agar gel immunodiffusion test, AGID) or are time consuming to perform (e.g., ELISA and western blotting). The development of a rapid and simple method for detecting the disease is therefore critical to control the spread of EIA. In this study, we designed and developed a colloidal gold immunochromatographic (GICG) test strip to detect antibodies against EIAV based on the double-antigen sandwich. Both the p26 and gp45 proteins were used as the capture antigens, which may help to improve the positive detection rate of the strip. We found that the sensitivity of the test strip was 8 to 16 times higher than those of two commercially available ELISA tests and 128 to 256 times higher than AGID, but 8 to 16 times lower than that of western blotting. The strip has good specificity and stability. When serum samples from experimental horses immunized with the attenuated EIAV vaccine (*n* = 31) were tested, the results of the test strip showed 100% coincidence with those from NECVB-cELISA and 70.97% with AGID. When testing clinical serum samples (*n* = 1014), the test strip surprisingly provided greater sensitivity and a higher number of “true positive” results than other techniques. Therefore, we believe that the GICG test strip has demonstrated great potential in the field trials as a simple and effective tool for the detection of antibodies against EIAV.

**Key points:**

*• A colloidal gold immunochromatographic (GICG) fast test strip was developed with good specificity, sensitivity* *,* *stability, and repeatability*

*• The test strip can be used in point-of-care testing for the primary screening of EIAV antibodies*

*• Both the p26 and gp45 proteins were used as the capture antigens, giving a high positive detection rate in the testing of experimentally infected animal and field samples*

## Introduction

Equine infectious anemia (EIA) is listed by the World Organization for Animal Health (WOAH) as a strictly notifiable disease, and is an infectious disease of horses caused by the equine infectious anemia virus (Arakawa et al. 1960). EIAV belongs to the family *Retroviridae,* subfamily *Orthoretrovirinae* and is a member of the genus *Lentivirus* (Cheevers and McGuire 1985). Acute symptoms of horses infected with EIAV include intermittent high fever, thrombocytopenia, hemorrhage, edema, and anemia (Clabough et al. 1991; Sellon 1993). After the acute phase, the replication of EIAV in horses can eventually be controlled to low levels by the host immune system. At this point, the infected animals show chronic and relapsing subclinical symptoms, which include emaciation and progressive weakness (Hammond et al. 2000, 1999; Issel et al. 1982). The virus always persists in its hosts during subclinical infection and can still be transmitted through blood contact or by hematophagous insect vectors. Therefore, in the absence of an effective vaccine and pathognomonic signs, the accurate and timely identification of equids with inapparent infection is a key step for the prevention and control of EIA epidemics (Borges et al. 2013; Cook et al. 2013; Issel et al. 1982, 2014; Issel and Coggins 1979).

Conventional virus isolation techniques are not easy to achieve for EIAV, as most of the virulent EIAV strains do not adapt readily to cell culture in vitro (Hines and Maury 2001; McGuire et al. 1971). Currently, the detection of viral nucleic acids and the detection of EIAV-specific antibodies are the major laboratory testing methods for case confirmation Because there is a “serological window,” where the first production of antibodies against EIAV is generally between 21 and 28 days after inoculation, nucleic acid detection is necessary for detection of the early stages and outbreak of the disease infection (Cook et al. 2013; Issel et al. 2013). The WOAH-recommended method based on amplifying a region of the EIAV gag has been widely adopted. However, the applicability and accuracy of this technique are limited because of the low levels of homology among geographically distinct EIAV viral isolates at the primer-binding region (Cook et al. 2019; Dong et al. 2012). Moreover, nucleic acid detection is not adequately sensitive to detect low viral loads in blood (Cook et al. 2003; Harrold et al. 2000). In most of the cases, equids infected with EIAV show serological reactions to viral structural proteins such as Gag and Env at the late acute infection and subclinical and inapparent phases. Consequently, serological tests that are able to measure antibodies in equine sera are considered to be the most reliable tool for the long-term monitoring of EIA.

The agar gel immunodiffusion test (AGID) was developed decades ago. This test is based on the presence of antibodies against the capsid protein p26, and is considered to be the “gold standard” for the international import and export trades as prescribed by the WOAH (Alvarez et al. 2007b; Tencza et al. 2000). The AGID test is inexpensive and has high specificity to EIA-infected animals. However, it requires significant operating experience and provides low sensitivity. Western blotting tests provide high sensitivity by comparison and are another official-standard assay for the diagnosis of EIA (Issel et al. 2013). However, both of these official tests require more than 24 h before the results can be obtained. Another technique, enzyme-linked immunosorbent assays (ELISA), has been developed and is based on the detection of antibodies against major viral structural proteins such as the capsid protein p26, surface glycoprotein gp90, or transmembrane glycoprotein gp45 (Hu et al. 2023; Jin et al. 2004; Singha et al. 2013). Compared with AGID, ELISA is more sensitive and time saving, but the commercial ELISA kits are expensive and need special laboratory facilities and skilled technical operators.

In recent years, there has been increasing interest in the application of colloidal gold immunochromatographic (GICG) test strips (Authier et al. 2001; Faulk and Taylor 1971; Yeo et al. 2017), as they are simple to operate, rapid, highly sensitive, and low in cost (Wan et al. 2022). In this study, we designed and developed a double-antigen-sandwich GICG test strip for the detection of antibodies against EIAV in equine sera.

The recombinant p26 and gp45 proteins were expressed and purified from engineering bacteria and used to develop a double-antigen-sandwich GICG test strip for the detection of EIAV antibodies in equine sera. The conjugated pad was coated with colloidal gold-specific proteins, the test line (T-line) was coated with p26 alone or both p26 and gp45 proteins as the capture proteins, and the control line (C-line) was coated with the monoclonal antibody (MAb) 1G11 against p26 protein. The sensitivity of the test was compared with those of AGID, western blotting, and two commercial ELISA kits. The specificity, stability, and reproducibility of the test were assessed. The performance of the test strip was further evaluated using 31 serum samples from experimental horses immunized with the attenuated EIAV vaccine, as well as with 1186 clinical serum samples.

## Materials and methods

### Plasmids and hybridoma cell

Recombinant plasmids pET-30a-*p26*, pET-32a-*gp45*, and hybridoma 1G11 cell were stored in our laboratory (Du et al. 2018; Hu et al. 2014). The resuscitated hybridoma 1G11 cell was injected into the abdominal cavities of mice to obtain monoclonal antibody (MAb) 1G11. Ascetic fluids were collected and purified using a protein G affinity column (GE Healthcare, Uppsala, Sweden) as previously described (Hu et al. 2016). The purified MAb 1G11 was confirmed using an ultraviolet spectrophotometer at 280 nm.

### Serum samples

One sensitivity control serum was stored in our laboratory and used to evaluate the analytical sensitivity of our test. Commercial sera positive for equine herpesvirus type 1 (EHV-1), 4 (EHV-4), equine influenza virus (EIV), and equine arteritis virus (EAV) were purchased from National Veterinary Services Laboratories (NVSL, Trent, UK). Positive sera against *Escherichia coli* (*E. coli*), *Streptococcus equi* (*S. equi*), *Salmonella* Abortusequi (*S*. Abortusequi), *Burkholderia mallei* (*B. mallei*), *Theileria equi* (*T. equi*), *Babesia caballi* (*B. cabcalli*), and *Trypanosoma evansi* (*T. evansi*) were stored in our laboratory. Thirty-one serum samples from experimental horses immunized with the attenuated EIAV vaccine (Lin et al. 2020) and the 1186 clinical serum samples were also stored in our laboratory. These included sera collected from the field from EIAV-positive sick horses confirmed as positive for EIAV-antibody using AGID (*n* = 5), sera positive for other pathogens (*n* = 166), sera taken from horses in Argentina and kindly provided by Dr. Maria Barrandeguy at the Institute of Virology (*n* = 139), and sera taken from healthy horses across China (*n* = 876). As shown as Table 1, 166 clinical sera positive for other pathogens were tested using a competitive enzyme-linked immunosorbent assay (cELISA), an indirect enzyme-linked immunosorbent assay (iELISA), a card agglutination test for trypanosomiasis (CATT), hemagglutination inhibition (HI), serum neutralization tests (SNT), the tube-agglutination test (TAT), and the complement fixation test (CFT), which are recognized as the standard test methods for the detection of pathogens in equine samples.

**Table 1:** Specificity of the test strip I for testing clinical equine serum samples

Serum samples	Detection method	Total no	No. samples testing positive with test strip I
*B. caballi*-positive	cELISA	9	0
*T. equi*-positive	cELISA	2	0
*T. evansi*-positive	CATT	65	0
EIV-positive	HI	19	0
EHV-1-positive	SNT	6	0
EHV-4-positive	SNT	2	0
EAV-positive	SNT	9	0
*S.* Abortusequi-positive	TAT	20	0
*S.* equi-positive	iELISA	7	0
*B.mallei*-positive	CF	27	0

### Production and purification of proteins

The *p26* or *gp45* gene carried on the recombinant plasmids pET-30a-*p26* or pET-30a-*gp45* was subcloned into a pET-32a vector to get the plasmids pET-32a-*p26* or pET-32a-*gp45*, respectively. The plasmids were then transformed separately into *E. coli* BL21 (Tsingke, Beijing, China) to overexpress the proteins with a His-tag at their N-terminal. Briefly, the transformed BL21 cells were induced at 25 °C with an IPTG (isopropyl-b-D-1-thiogalactopyranoside) concentration of 0.5 mM for 8 h. The solubilized His-p26 (44.64 KDa) and His-gp45 (32.50 KDa) proteins were then purified using HisPur™ Ni–NTA Resin (Thermo Fisher Scientific, MA, USA) according to the manufacturer’s protocol and as described previously (Du et al. 2018; Hu et al. 2014). In order to eliminate cross-reactions and to improve the specificity of the reaction, the His-tags were then removed from the N-terminal of the His-p26 and His-gp45 proteins using the thrombin digestion method. Briefly, the His-p26 or His-gp45 proteins were treated with 0.5-NIH units/mg thrombin protein from bovine plasma (Beyotime, Shanghai, China) at 4 °C for 12 h under constant agitation. Bestatin (Beyotime, Shanghai, China), aprotinin (Solarbio, Beijing, China), leupeptin (Yuanye, Shanghai, China), and cysteine protease inhibitor (Yuanye, Shanghai, China) were then added simultaneously to final concentrations of 3 mg/mL, 2 mg/mL, 5 mg/mL, and 5 mg/mL, respectively. HisPur™ Ni–NTA Resin was equilibrated with 20 mM PB three times and was loaded onto an affinity chromatography column tube (Beyotime, Shanghai, China). The protein solution was then loaded onto the column tube at 4 °C for 5 min under constant agitation. The flowthrough solution containing the recombinant p26 (30.72 KDa) and gp45 (18.58 kDa) proteins was collected and was analyzed with both sodium dodecyl sulfatepolyacrylamide gel electrophoresis (SDS-PAGE) and western blotting. Western blotting was performed as follows. Briefly, 0.10 μg of His-p26 and His-gp45 proteins was subjected to 12% SDS-PAGE, and then transferred onto NC membranes. The membranes were blocked with 5% nonfat dry milk in 0.01 M PBS saline for 2 h and were probed with EIAV-positive serum or mouse antibody against His-tag at a dilution of 1:1000 for 2 h at room temperature. The membranes were then incubated with either HRP-labeled anti-horse IgG (KPL, Hemet, USA) or biotin-labeled anti-mouse IgG (KPL, Hemet, USA) at a dilution of 1:10,000 for 1 h. Protein bands were detected using an HRP-DAB Substrate Colorimetric Kit (Solarbio, Beijing, China) or an Odyssey infrared imaging system (LI-COR, UK) at a wavelength of 800 nm. The protein immunogenicity was checked by verification with positive and negative sera, and samples from clinically equivocal sera were then tested with western blotting test to confirm their status.

## Preparation of conjugate pad with colloidal gold p26 and colloidal gold gp45

Colloidal gold solutions containing gold particles with an average diameter of 20 nm were prepared according to the citrate reduction method (Wan et al. 2022; Yang et al. 2022; Zou et al. 2021). Briefly, 1 mL of chloroauric acid (HAuCl_4_, 1%) was added to about 100 mL of boiling water under constant agitation, and 1 mL of 1% sodium citrate aqueous solution was then added when the solution had reached boiling again. After the mixture had changed color to wine red, it was allowed to boil for a further 5 min. Finally, 0.1 mL of 0.05% sodium azide was added as a preservative, and water was added to make up the volume to exactly 100 mL. In order to determine the amount of recombinant protein conjugated with the colloidal gold, the samples were tested using cuvette observation (Bai et al. 2022). The optimal conjugated amount of protein was 9.6 µg/mL, which was 150% of the minimum chromogenic amount of protein showing an invariable color. Finally, the solution was sprayed onto a treated glass fiber membrane (conjugate pad) at 2.5 μL/cm and placed in a drying oven at a humidity of 10–30% and a temperature of 37 °C for 12 h.

### Coating of test line (T-line) and control line (C-line)

PBS buffer (0.01 M, pH 7.4) was used as a coating solution to dilute the proteins (p26/gp45) to 0.4 mg/mL, and Tris–HCl (0.02 M, pH 8.6) was used to dilute the MAb 1G11 to 1.0 mg/mL. The NC membrane was attached to the polyvinyl chloride plate (PVC plate) with backing adhesive, and then the conjugate pad was sprayed with the diluted proteins and MAb separately as the T-line and C-line on the nitrocellulose membrane (NC membrane; Merck-Millipore, MA, USA) using XYZ Platform Dispenser (HM3030; Kinbio, Shanghai). Finally, both were placed in a drying oven with humidity of 10–30% and at a temperature of 37 °C for 12 h.

### Optimization of the GICG test strip

If no protein, or insufficient protein, is added to each tube of colloidal gold solution, an agglomeration occurs, changing the red color to blue. The amount of recombinant protein that can maintain the red color of the colloidal gold solution unchanged can be determined, indicating the quantity of recombinant protein bound to the colloidal gold. In our case, this was 9.6 µg/mL. We then adjusted the pH of the colloidal gold solution using 0.2 mol/L K_2_CO_3_ or 0.1 mol/L HCl to determine the optimal pH value for labeling proteins with colloidal gold, which we found to be 6. We used this information to optimize the protein coating concentrations for the T- and C-lines. Recombinant protein and MAb were then separately diluted to different concentrations and applied to the NC membrane in lines. The optimal concentration of gold-labeled mAb was determined based on the intensity of the bands on the T-line and C-line and the background color of the NC membrane. The optimal concentrations were found to be 0.4 mg/mL for p26/gp45 and 1.0 mg/mL for MAb 1G11. Finally, to allow standardization of the method and to reduce background interference, we selected the best matching solutions from a variety of commercial conjugated pads, sample pads, NC membranes, and reagents.

### Production of the GICG test strip

The sample pad, the conjugated pad coated with colloidal gold-proteins, the NC membrane coated with the capture proteins (T-line) and the MAb 1G11 (C-line), and the absorbent pad were adhered to a PVC plate in the proper order. The conjugated pad and the NC membrane (T-line) of GICG test strip I were coated with p26 and gp45, while GICG test strip II was coated with p26 protein alone. The assembled membranes were packed into a case, wrapped in foil bags, and stored at 4 °C.

**Fig. 1:**
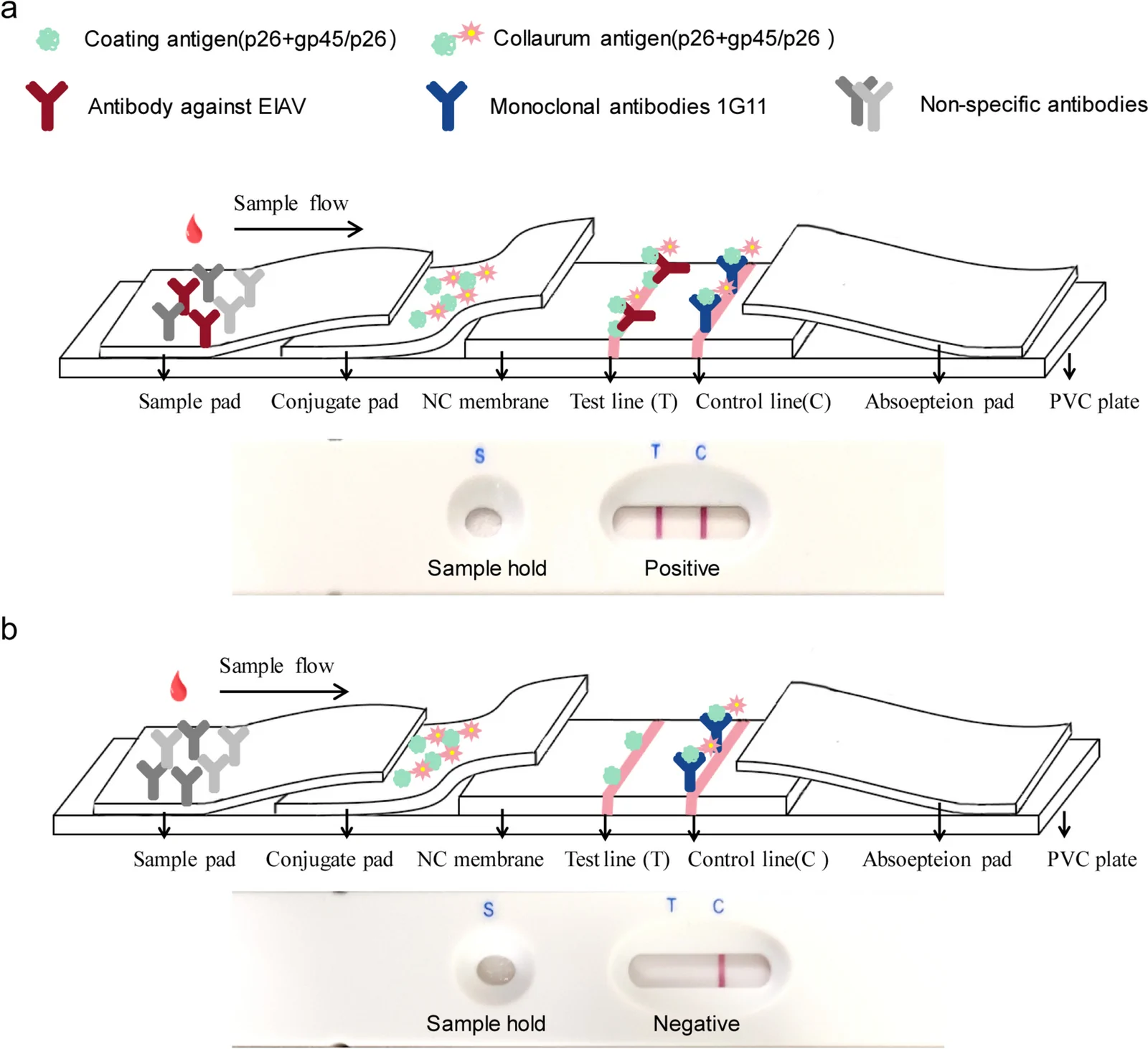
Schematic interpretation of the colloidal gold test strip. Every test strip included three pads (sample pad, conjugate pad, and absorbent pad), a nitrocellulose membrane, and a polystyrene backing board. The conjugate pad of the test strip contained either gold-labeled p26 and gp45 (test strip I) or gold-labeled p26 (test strip II), which provided purple-red colors. There were two lines on the nitrocellulose membrane: the test line (T-line) and the control line (C-line). The T-line of the test strip was coated with either p26 and gp45 (test strip I) or p26 alone (test strip II). The C-line was coated with monoclonal antibody (MAb) 1G11 against EIAV. **A** If the serum samples contain antibodies against EIAV, the specific EIAV-IgGs would bind with colloidal gold-antigens, and the conjugates would subsequently react with the proteins coated on the T-line after migrating into the NC membrane by capillary action. In this case, a red–purple band emerges on the T-line. Unbound colloidal gold-protein reacts with the MAb 1G11 at the C-line to form a purple-red band. **B** If the serum samples do not contain antibodies against EIAV, unbound colloidal gold-protein runs over the T-line and then reacts with the MAb 1G11 at the C-line to form a purple-red band. In this case, no red–purple band occurs at the T-line

Samples containing 10 μL serum and 100 μL sample buffer (0.01 M PBS, 0.05% Tween-20) were added to the sample pad. As shown in the schematic interpretation (Fig. 1), the serum migrates from the sample pad toward the conjugate pad, where it reacts with the microparticles and moves through the NC membrane. If the serum contains EIAV antibody, a red–purple band (readily visible to the unaided eye) emerges at the T location. Our test results were read between 10 and 15 min after the addition of the sample. The result was considered positive if a purplish-red band appeared at both the C- and T-lines, and was considered negative if no line appeared on the T-line. The C-line is a crucial control of product quality. If there was no band on the C-line, we considered that there was a problem with the experiment and the result from that strip was invalid.

### Evaluation of the sensitivity, specificity, stability, and reproducibility of the GICG test strip

To evaluate the analytical sensitivity of the resulting GICG strip, a two-fold serial dilution of a sensitivity control serum sample was generated using 0.01 M PBS (dilutions ranged from 1:2^0^ to 1:2^14^). The serial dilution was tested using test strip I, test strip II, AGID, two cELISA kits (IDEXX, Westbrook, USA; NECVB, Harbin, China), and western blotting. The two ELISAs were conducted according to the instructions in the kits, the AGID test developed in our lab referred to the standards formulated by WOAH, and the western blotting was performed as described previously (Hu et al. 2016). Meanwhile, five field samples of EIAV-positive sera from sick horses were used for the assessment of test sensitivity. The analytical specificity of the strips was determined by testing 166 clinical serum samples positive for different pathogens. These sera included those positive for equine viruses (EHV-1,4, EIV, EAV), bacteria (*E. coli*, *S. equi*, *S. abortusequi*, and *B. mallei*) and parasites (*T. equi*, *B. caballi*, and *T. evansi*), as well as a serum negative for all parasites, viruses, and bacteria collected from a healthy horse. Test strip I was stored at 37 °C for 3 days, 7 days, 14 days, or 21 days, and at 25 °C for 6 months to check the stability of the test, and was assessed using diluted sera positive for EIAV. Each diluted serum sample was tested five times to assess the reproducibility of the results.

### Western blotting

In order to determine whether the equivocal serum samples (test strip I positive but AGID and/or NECVB cELISA negative) were “true positives” according to the presence of EIAV-antibodies, a western blotting was performed as described previously (Alvarez et al. 2007a). Briefly, 0.10 μg of purified 30a-His-p26 (33.13 KDa) and His-gp45 (32.50 KDa) proteins was subjected to 12% SDS-PAGE. Following SDS-PAGE, the separated proteins were transferred onto NC membranes. The membranes were blocked with 5% nonfat dry milk in 0.01 M PBS saline for 2 h and were probed with equivocal serum samples at a dilution of 1:1000 for 2 h at room temperature. The membranes were then incubated with anti-horse IgG and peroxidase labeled HRP (KPL, Hemet, USA) at a dilution of 1:10,000 for 1 h. Protein bands were detected using an HRP-DAB substrate colorimetric kit (Solarbio, Beijing, China).

## Results

### Expression and purification of EIAV p26 and gp45 proteins

**Fig. 2:**
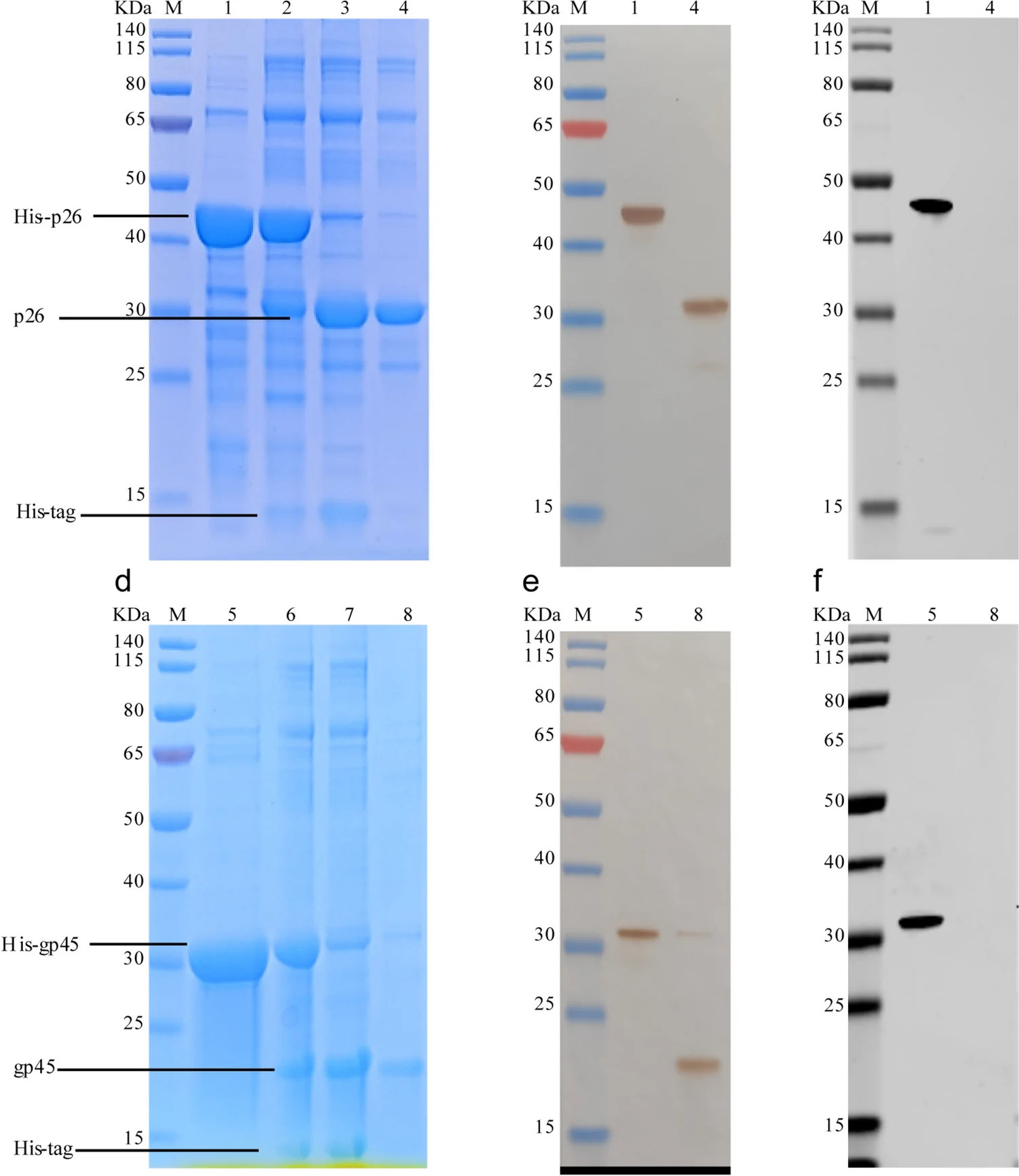
SDS-PAGE and western blot analysis of purified capture protein. **A** Purification of p26 protein with and without His-tag. Lanes: M, protein marker; 1, purified His-p26 protein; 2, His-p26 protein cleaved by thrombin for 3 h; 3, His-p26 protein cleaved by thrombin for 12 h; 4, purified p26. **B** Western blotting detection of p26 protein with and without His-tag by EIAV-positive serum. **C** Western blotting detection of p26 protein with and without His-tag by mouse antibody against His-tag. **D** Purification of gp45 protein with and without His-tag. Lanes: M, protein marker; 5, purified His-gp45 protein; 6, His-gp45 protein cleaved by thrombin for 3 h; 3, His-gp45 protein cleaved by thrombin for 12 h; 4, purified gp45. **E** Western blotting detection of gp45 protein with and without His-tag by EIAV-positive serum. **F** Western blotting detection of gp45 protein with and without His-tag by mouse antibody against His-tag

The constructed prokaryotic expression plasmids pET-32a-*p26* and pET-32a-*gp45* were transformed into *E. coli* BL21 (DE3) for induction. The purified His-p26 and His-gp45 proteins were separately treated with thrombin at 4 °C for 12 h with constant agitation. Solutions were then combined with HisPur™ Ni–NTA resin at 4 °C for 5 min and subjected to affinity chromotography. The purified p26 and gp45 proteins could be found in the flow fluid. These purified proteins could be detected with antibodies against EIAV, but antibodies against the His-tag did not react with them (Fig. 2).

### Sensitivity analysis of the GICG test strip

**Fig. 3:**
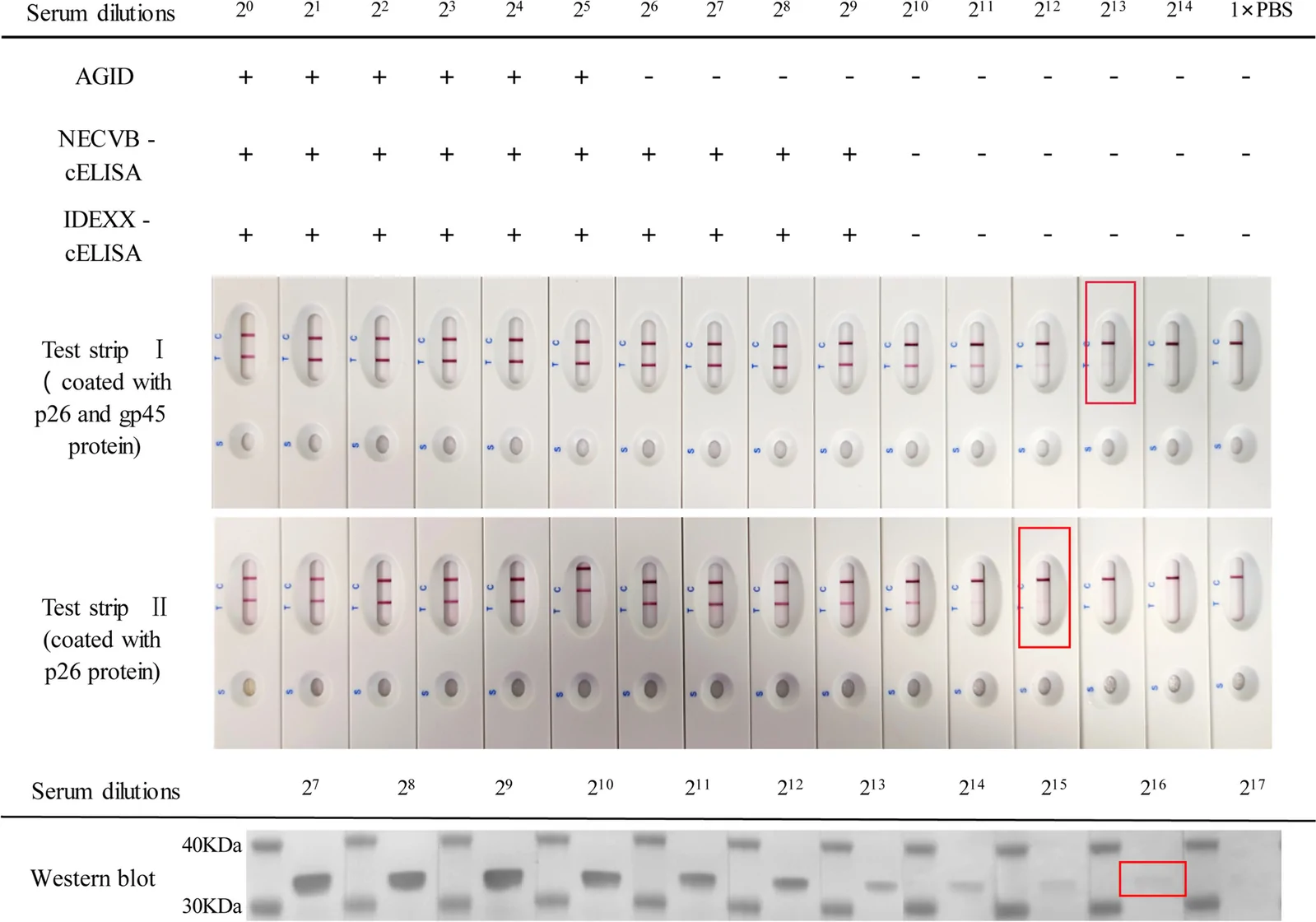
Analytical sensitivity of the test strip and comparison with AGID, ELISA, and western blotting. The sensitivity was evaluated using serial dilutions of the EIAV sensitivity control serum. The minimum detection limitation of AGID, NECVB-cELISA, IDEXX-cELISA, test strip I, test strip II, and western blotting was 1:2^5^, 1:2^9^, 1:2^9^, 1:2^13^, 1:2^12^, and 1:2^16^ dilution, respectively. Note: “ + ,” positive; “-,” negative

The serially diluted EIAV sensitivity control serum could be determined as positive using AGID down to a dilution of 1:2^5^ dilution. The serially diluted EIAV serum was then also used to assess test strip I, strip II, IDEXX-cELISA, NECVB-cELISA, and western blotting, and the minimum detection limits of these techniques were found to be dilutions of 1:2^13^, 1:2^12^, 1:2^9^, 1:2^9^, and 1:2^16^, respectively (Fig. 3). We found that the sensitivity of test strip I was higher than that of test strip II, and therefore we selected test strip I for the subsequent experiments. Test strip I showed a positive result for all five of the EIAV-positive field sera, demonstrating that its detection sensitivity was 100%.

### Specificity analysis of the GICG test strip

**Fig. 4:**
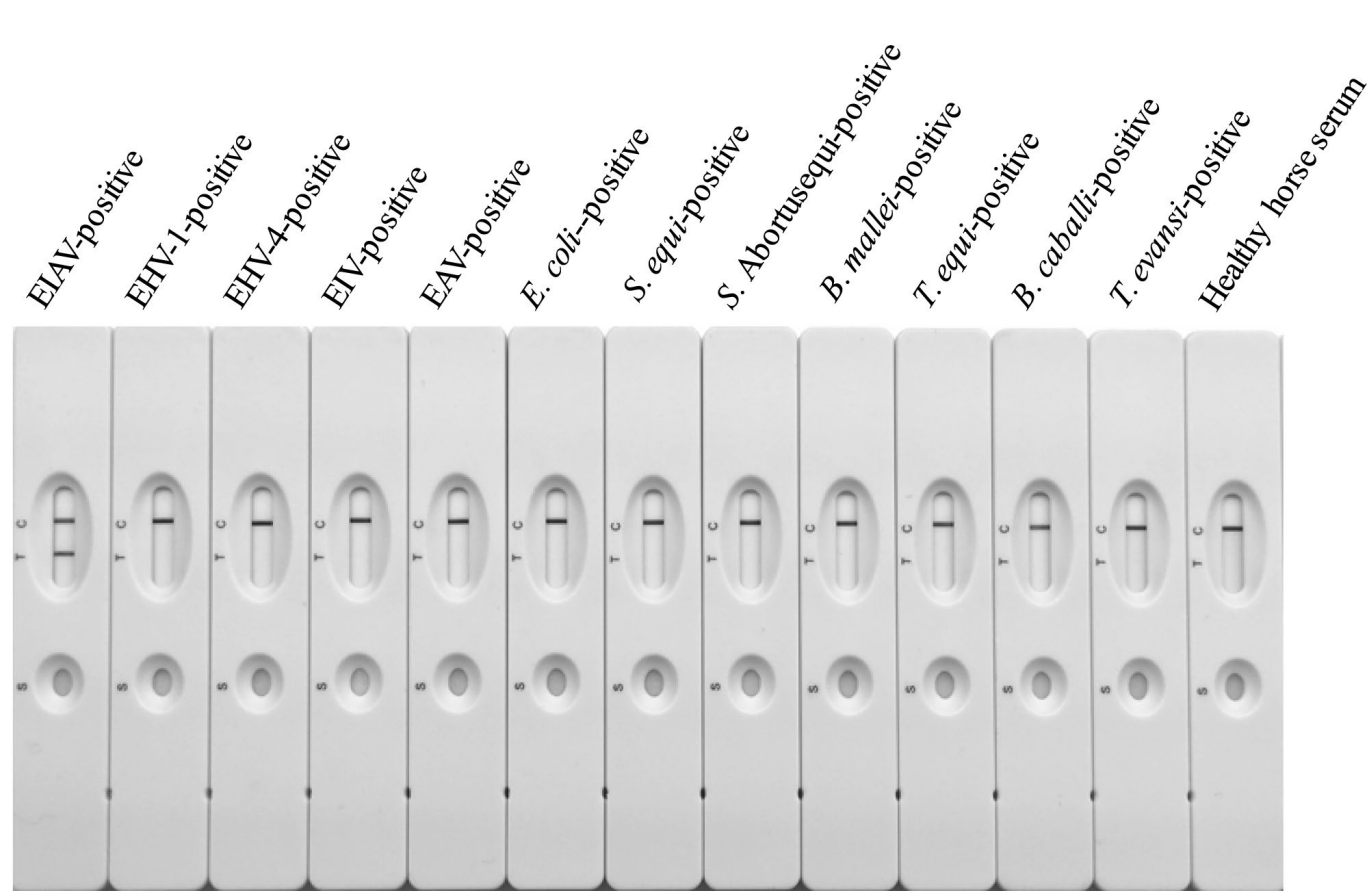
Analytical specificity of test strip I. Test strip I can only detect antibodies against EIAV, but not those against other equine viruses (EHV-1,4, EIV, EAV), bacteria (*E. coli*-, *S. equi*, *S. abortusequi*, *B. mallei*), or parasites (*T. equi*, *B. caballi*, *T. evansi*). Test strip I did not detect antibodies in the serum taken from a healthy horse

Test strip I was only able to detect antibodies against EIAV, but not those specific to other equine viruses (EHV-1,4, EIV, EAV), bacteria (*E. coli*, *S. equi*, *S. abortusequi*, and *B. mallei*), or parasites (*T. equi*, *B. caballi*, and *T. evansi*), and the serum taken from a healthy horse also tested negative (Fig. 4). There was no cross-reactivity with any of the 166 clinical serum samples positive for pathogens when tested with test strip I (Table 1), demonstrating the detection specificity of this test.

### Stability of the GICG test strip and reproducibility of results

Test strip I was stored at 37 °C for 3 days, 7 days, 14 days, or 21 days, at 25 °C for 6 months, and then was tested with the sensitivity control serum (repeated five times). The color intensities of the resulting bands in most of the processed strips were highly consistent with those of the new strip. Strip I thus is stable and remains functional at room temperature (25 °C) for at least half a year (Table 2), and the results from the strip are repeatable.

**Table 2:** Stability and reproducibility of the test strip I

Serum dilution	1/2^7^	1/2^8^	1/2^9^	1/2^10^	1/2^11^	1/2^12^	1/2^13^	1/2^14^
New strips	+ (5/5)	+ (5/5)	+ (5/5)	+ (5/5)	+ (5/5)	+ (5/5)	+ (5/5)	− (5/5)
37 ℃ for 3 days	+ (5/5)	+ (5/5)	+ (5/5)	+ (5/5)	+ (5/5)	+ (5/5)	+ (5/5)	− (5/5)
37 ℃ for 7 days	+ (5/5)	+ (5/5)	+ (5/5)	+ (5/5)	+ (5/5)	+ (5/5)	+ (5/5)	− (5/5)
37 ℃ for 14 days	+ (5/5)	+ (5/5)	+ (5/5)	+ (5/5)	+ (5/5)	+ (5/5)	+ (4/5)	− (5/5)
37 ℃ for 21 days	+ (5/5)	+ (5/5)	+ (5/5)	+ (5/5)	+ (5/5)	+ (4/5)	+ (2/5)	− (5/5)
25 ℃ for 6 months	+ (5/5)	+ (5/5)	+ (5/5)	+ (5/5)	+ (5/5)	+ (5/5)	+ (5/5)	− (5/5)

### Comparison of results from test strip, AGID, and cELISA

As shown in Table 3, 31 serum samples from experimental horses immunized with the attenuated EIAV vaccine were tested using test strip I, AGID, and a cELISA kit (NECVB, Harbin, China). The results from test strip I and the NECVB-cELISA were identical, with a coincidence rate of 100%, although the coincidence rate between test strip I and AGID was low (70.91%). One thousand and fifteen clinical serum samples from horses in China (*n* = 876) and Argentina (*n* = 139) were also tested using the above three methods. As shown in Table 4, 100% of the samples testing positive with AGID and NECVB-cELISA also tested positive with strip I. Similarly, of those samples testing negative with the AGID or NECVB-cELISA methods, 98.97% (964/975) and 99.38% (964/970), respectively, also tested negative using strip I. The overall rate of coincidence of the test strip I with AGID was 98.92% (1004/1015) and was 99.41% (1009/1015) with NECVB-cELISA.

**Table 3:** Comparison of the results for test strip I with AGID and NECVB-cELISA for testing 31 serum samples from experimental horses immunized with the attenuated EIAV vaccine

		AGID			NECVB-cELISA		
		Positive	Negative	Total	Positive	Negative	Total
Test strip I	Positive	22	9	31	31	0	31
	Negative	0	0	0	0	0	0
	Total	22	9	31	31	0	31
	The coincidence rate			70.97%			100%

**Table 4:** Comparison of the results for test strip I with AGID and NECVB-cELISA for testing 1015 clinical serum samples

		AGID			NECVB-cELISA		
		Positive	Negative	Total	Positive	Negative	Total
Test strip I	Positive	40	11	51	45	6	51
	Negative	0	964	964	0	964	964
	Total	40	975	1015	0	970	1015

### Confirmation of equivocal sera with western blotting

**Fig. 5:**
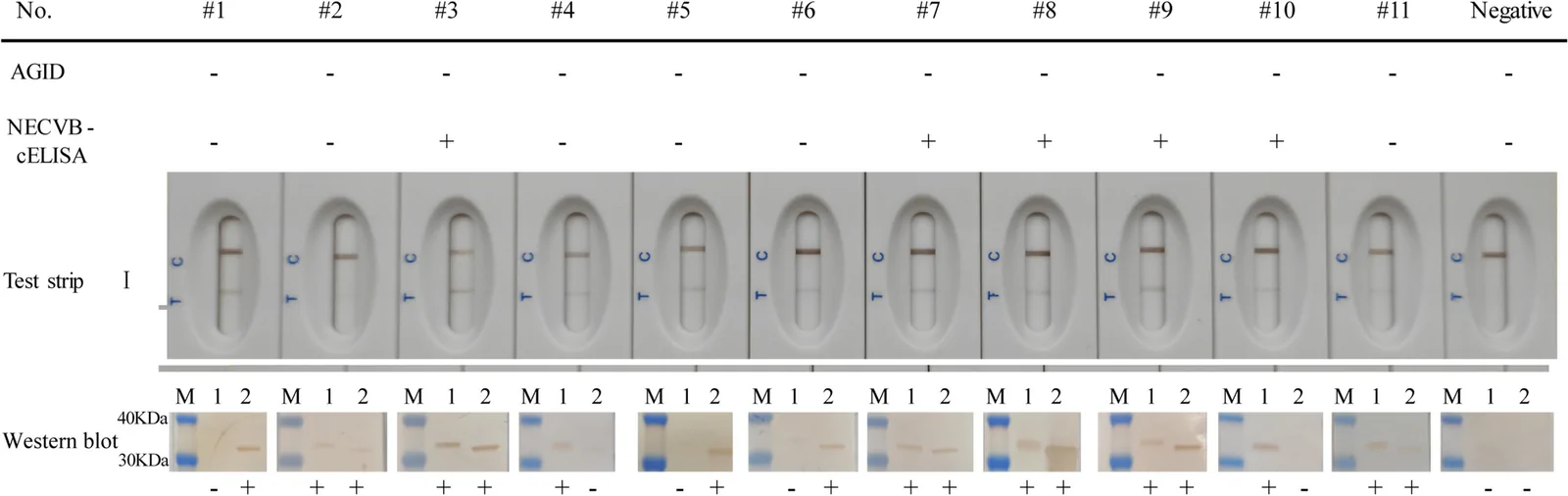
Results for 11 equivocal sera from AGID, NECVB-cELISA, test strip I, and western blotting. All results from test strip I and the western blotting were positive except for those from the negative serum. Lanes: M, protein marker; 1, 30a-His-p26; 2, His-gp45. Note: “ + ,” positive; “ − ,” negative

There were 11 equivocal sera (Table 4). These included five samples which tested positive with test strip I and NECVB-cELISA, but negative for AGID, and six samples that tested positive with test strip I but negative for both AGID and NECVB-cELISA. In order to confirm the status of these equivocal sera, these 11 clinical samples were subjected to western blotting, targeting the 30a-His-p26 protein and His-gp45 protein of EIAV. As shown in Fig. 5, specific bands confirming the presence of the antibodies against EIAV p26 protein or/and gp45 were obtained from each serum, demonstrating that equivocal sera all contained antibodies against EIAV. This shows that test strip I did not produce false positive results.

## Discussion

Equine infectious anemia (EIA) was first described in 1843 and was confirmed as having a viral etiology in 1904 (Cheevers and McGuire 1985). Today, EIA is a widespread disease resulting in considerable harm to equine industries worldwide (Cook et al. 2013; Machado et al. 2021). EIA is a blood-borne infectious disease caused by the equine infectious anemia virus (EIAV), which belongs to the genus *Lentivirus* (*Retroviridae*) (Hu et al. 2016; Sellon 1993). With increasing internationalization, horses are being frequently moved around the world. Consequently, the importance of timely and consistent monitoring of equine diseases cannot be overstated. Large numbers of studies have demonstrated that the EIA virus persists in hosts, progressing from the acute stage to a prolonged asymptomatic period. This results in hosts becoming potential reservoirs of EIAV transmission, or suffering relapses of the disease, following immune suppression of the virus (Borges et al. 2013; Issel et al. 1982; Issel and Coggins 1979).

In recent years, colloidal gold immunochromatographic (GICG) test strips have become more popular as serological tests (Authier et al. 2001; Faulk and Taylor 1971; Yeo et al. 2017), because of their numerous advantages, including ease of operation, rapidity, high sensitivity, and low cost (Wan et al. 2022). In this study, we designed and developed a double-antigen-sandwich GICG test strip based on recombinant p26 and gp45 proteins without their 6 × His-tags, for the detection of EIAV antibodies in equine sera. If the serum samples contain antibodies against EIAV, the specific EIAV-IgGs binds with colloidal gold-p26 or colloidal gold-gp45. The conjugates subsequently react with the p26- or gp45-coated T-line after migrating into a NC membrane by capillary action. A positive result leads to the emergence of a red–purple band. Unbound colloidal gold-p26 runs over the T-line and reacts with the anti-p26 antibody (MAb 1G11) at the C-line to form a purple-red band, irrespective of whether the serum sample contains antibodies against EIAV.

Our study contains some slight differences from previously published GICG methods (Alvarez et al. 2010; Bai et al. 2022; Chu et al. 2005; Costa et al. 2016; Zhao et al. 2017). Firstly, we improved the sensitivity of the test reaction by optimizing the reaction conditions. Furthermore, polyhistidine tag (His-tag) expression systems are widely applied as fusion expression systems because they facilitate protein expression and simplify protein purification (Khoo et al. 2005; Meredith et al. 2004). It generally believed that separation of the His-tag from the target protein is unnecessary on account of the low molecular weight of the tag and because it does not affect protein structure or functions (Mason et al. 2001). However, with the development of engineered vaccines, especially recombinant protein vaccines, the insertion of a His-tag onto the target protein is potentially an issue due to cross-reactions caused by the tags (Booth et al. 2018; Paul et al. 2020; Zhao and Huang 2016). To improve the specificity of our test, we used target proteins without a 6 × His-tag. Unfortunately, we have not done further research on the effect of the His-tag on antigen–antibody reactions, and more data are still needed in this field.

The nucleotide sequence encoding viral core protein p26 produces an immune response in approximately 89% of test cases (Cook et al. 2013), and is therefore used as a capture protein in most research into tests for the detection of antibodies against viral proteins. Specific antibodies against the p26 protein can usually be detected between 14 and 45 days following infection, and those against gp45, between about 10 and 30 days after initial exposure (Coggins et al. 1972; Russi et al. 2023). The test strips in our study detected antibodies against both p26 and gp45 proteins, improving the positive detection rate (Fig. 5). Furthermore, as shown in Fig. 5, certain samples yielded negative results in the cELISA assay, while producing positive outcomes in the test strips and western blots. This observation implies that the colloidal gold test strips show heightened sensitivity in the detection of the p26 protein when compared to cELISA. This disparity can perhaps be attributed to the fluid dynamics in the colloidal gold method being less likely to disrupt the binding between the antigen and antibodies than the washing steps employed in cELISA. Where antibody levels are low, the cELISA washing process may lead to the loss of sufficient bound antibodies to bring the sample below the positive detection level of the test.

The test strip was found to have good specificity, high sensitivity, and was stable over half a year. The sensitivity of the test strip was 8 to 16 times higher than that of the two commercially available ELISA tests, and 128 to 256 times higher than that of AGID, although the test sensitivity was still lower than that of western blotting. When serum samples from experimental horses immunized with attenuated EIAV vaccine (*n* = 31) were tested, the results of the test strip showed 100% coincidence with the results of ELISA, although only a low rate of coincidence with the results of AGID (70.97%). Lower antibody concentrations in the serum samples and the low sensitivity of AGID may be the causes of false negative results from the AGID tests, while the same samples tested positive using the more sensitive ELISA and test strips. When clinical serum samples (*n* = 1014) were tested, western blot analysis confirmed the presence of the antibodies against EIAV p26 protein or/and gp45 in the equivocal sera (test strip I positive but NECVB-cELISA and/or AGID negative). These results demonstrated that our test strip I had, surprisingly, greater sensitivity and resulted in more “true positives” than the other commercially available tests. As shown in Fig. 5, the positive results from the test strip may be a result of the presence of gp45 antibodies (#1, #5, #6), which are produced earlier than those against p26 after viral infection. These anti-gp45 antibodies are detected by neither the AGID nor the cELISA tests, where the p26 protein alone was applied as the detection antigen. The GICG test strip is therefore a more sensitive and specific test than either AGID or NECVB-cELISA, and could be a reliable tool for clinical serum screening.

It is difficult to quantify the antibodies in serum samples, and the relationship between the presence of antibodies and the risk of relapse has not been studied. Furthermore, due to the limitations of prokaryotic expression system and purification process, the purified recombinant proteins may contain limited non-specific proteins of *E. coli*, which may lead to a risk of false positive results. Therefore, we suggest that the test strip be used as a simple tool for clinical screening, and that a final result should be confirmed by combining this technique with ELISA, AGID, and western blotting.

Compared with conventional laboratory detection methods, GICG test strips do not require sophisticated laboratory equipment or expensive instruments. Many of the vast but remote horse-breeding areas have no professional laboratory nearby or have only very basic laboratories. GICG test strips offer the possibility of on-site testing, saving costs and time as it is not necessary to transport the tests to professional laboratories. Commercial products come with a dropper and with 100-μL diluent that has been sub-packed into an EP tube. A drop of serum is added to the diluent and mixed, then dropped into the sample hole. Validity is checked automatically. In this way, the GICG test strip is highly suitable for use by veterinarians conducting preliminary screening against EIA during field testing. In conclusion, the results of the above analyses show that our test strips are simple to operate and provide rapid analysis and high sensitivity at low cost, meaning that they may be a powerful tool for the detection of EIAV antibodies and the monitoring of EIA.

## Data Availability

The data used to support the findings of this study are included within the article.
